# Supplementary material for: An open-source framework for large-scale, flexible evaluation of biomedical text mining systems
Source: J Biomed Discov Collab. 2008 Jan 29;3:1. doi: 10.1186/1747-5333-3-1 (PMC2276192; doi:10.1186/1747-5333-3-1)
Supplement: Additional file 1 — Evaluation platform source code. The additional file contains the software evaluation framework discussed in this paper. The most current release can be downloaded from SourceForge.net [1]. See the accompanying README file for instructions on installation and use. Also included in the distribution is a collection of UIMA wrappers for some commonly used BioNLP tools and annotated corpora. [file 1747-5333-3-1-S1.zip › bionlp_uima_repository-1.0.1/docs/api/edu/uchsc/ccp/util/nlp/annotation/Span.html]

Span (Center for Computational Pharmacology Util/UIMA API)


|  |  |  |
| --- | --- | --- |
| **Overview** \| **Package** \| **Class** \| **Tree** \| **Deprecated** \| **Index** \| **Help** | |  |
| **PREV CLASS**   **NEXT CLASS** | **FRAMES**    **NO FRAMES**     **All Classes** |  |
| SUMMARY: NESTED \| FIELD \| CONSTR \| METHOD | DETAIL: FIELD \| CONSTR \| METHOD |  |


---


## edu.uchsc.ccp.util.nlp.annotation Class Span

```
java.lang.Object
  edu.uchsc.ccp.util.nlp.annotation.Span
```

**All Implemented Interfaces:**: Comparable

---

``` public class Span extends Object implements Comparable ```

---

**Constructor Summary**

`Span(int spanStart,
int spanEnd)`


| **Method Summary** | |
| --- | --- |
| `Span` | `clone()` |
| `int` | `compareTo(Object spanToEquate)` |
| `boolean` | `endsBefore(Span span)`             Returns true if this span ends before the inputted span, false otherwise. |
| `boolean` | `equals(Object spanToEquate)`             The default equals() method uses the StrictSpanComparator |
| `int` | `getSpanEnd()` |
| `int` | `getSpanStart()` |
| `int` | `hashCode()` |
| `int` | `length()`             Return the length of the span |
| `boolean` | `overlaps(Span overlappingSpan)`             The overlaps() method uses the SloppySpanComparator |
| `void` | `setSpanEnd(int spanEnd)` |
| `void` | `setSpanStart(int spanStart)` |
| `boolean` | `startsBefore(Span span)`             Returns true if this span starts before the inputted span, false otherwise. |
| `String` | `toString()` |

**Methods inherited from class java.lang.Object**

`finalize, getClass, notify, notifyAll, wait, wait, wait`

**Constructor Detail**

### Span

```
public Span(int spanStart,
            int spanEnd)
     throws InvalidSpanException
```

**Throws:**: `InvalidSpanException`


**Method Detail**

### getSpanEnd

```
public int getSpanEnd()
```

---


### setSpanEnd

```
public void setSpanEnd(int spanEnd)
                throws InvalidSpanException
```

:   **Throws:**: `InvalidSpanException`

---


### getSpanStart

```
public int getSpanStart()
```

---


### setSpanStart

```
public void setSpanStart(int spanStart)
                  throws InvalidSpanException
```

:   **Throws:**: `InvalidSpanException`

---


### length

```
public int length()
```

:   Return the length of the span

    :   **Returns:**

---


### equals

```
public boolean equals(Object spanToEquate)
```

:   The default equals() method uses the StrictSpanComparator

    :   **Overrides:**: `equals` in class `Object`

---


### overlaps

```
public boolean overlaps(Span overlappingSpan)
```

:   The overlaps() method uses the SloppySpanComparator

    :   **Parameters:**: `overlappingSpan` - **Returns:**

---


### startsBefore

```
public boolean startsBefore(Span span)
```

:   Returns true if this span starts before the inputted span, false otherwise. (Equal span starts result in false).

    :   **Parameters:**: `span` - **Returns:**

---


### endsBefore

```
public boolean endsBefore(Span span)
```

:   Returns true if this span ends before the inputted span, false otherwise. (Equal span ends result in false).

    :   **Parameters:**: `span` - **Returns:**

---


### hashCode

```
public int hashCode()
```

:   **Overrides:**: `hashCode` in class `Object`

---


### toString

```
public String toString()
```

:   **Overrides:**: `toString` in class `Object`

---


### clone

```
public Span clone()
```

:   **Overrides:**: `clone` in class `Object`

---


### compareTo

```
public int compareTo(Object spanToEquate)
```

:   **Specified by:**: `compareTo` in interface `Comparable`


---


|  |  |  |
| --- | --- | --- |
| **Overview** \| **Package** \| **Class** \| **Tree** \| **Deprecated** \| **Index** \| **Help** | |  |
| **PREV CLASS**   **NEXT CLASS** | **FRAMES**    **NO FRAMES**     **All Classes** |  |
| SUMMARY: NESTED \| FIELD \| CONSTR \| METHOD | DETAIL: FIELD \| CONSTR \| METHOD |  |


---
